# CD40LG Downregulation in Lung Adenocarcinoma: A Prognostic Biomarker Linked to Immune Cell Infiltration and Survival Outcomes

**DOI:** 10.7150/jca.115525

**Published:** 2025-08-22

**Authors:** Peng Tang, Ruihao Zhang, Yuqi Zhang, Yuan Ao, Yanan Wang, Guannan Wang, Wenjun Wang, Junmei Xu, Fang Hu, Guangsheng Zhu

**Affiliations:** 1 Key Laboratory of Digestive Cancer of Tianjin, Tianjin's Clinical Research Center for Cancer, National Clinical Research Center for Cancer, Tianjin Medical University Cancer Institute and Hospital, Tianjin 300060, China.; 2 Tianjin Medical University General Hospital, Tianjin 300052, People's Republic of China.; 3 Department of Thoracic Surgery, Shanghai Tongji Hospital, School of Medicine, Tongji University, Xincun Rd. 389, Shanghai, 200065, People's Republic of China.; 4 Gomics Gene Technology Co. Ltd, Hangzhou, 310000, China.

**Keywords:** CD40LG, Immune Microenvironment, LUAD, Biomarker, Immunotherapy Biomarkers.

## Abstract

**Background:** Lung adenocarcinoma (LUAD) continues to pose a major challenge in cancer treatment, characterized by low survival rates, particularly in metastatic instances. Although immunotherapy has become a common approach for treating LUAD, its success rate is merely 20%, hindered by the absence of effective biomarkers. This limitation is likely influenced by the tumor's immune microenvironment. CD40LG, an important immune molecule, has emerged as a crucial factor in modulating the tumor immune environment and affecting the outcomes of immunotherapy. Nevertheless, its exact mechanisms in LUAD remain unclear, requiring more research.

**Methods:** Through a correlation analysis of prognosis and clinical traits across multiple cancers, we determined that CD40LG is significant in LUAD. This role was confirmed using various public datasets. We evaluated CD40LG protein expression in 94 LUAD and nearby non-cancerous tissues via immunohistochemistry. To gauge immune cell infiltration, we employed multiplex immunofluorescence staining on tissue microarrays. Additionally, *in vitro* assays were carried out to explore the effects of CD40LG modulation on the behavior of LUAD cells.

**Results:** Various cancers, including LUAD, exhibited down-regulation of CD40LG, which correlated with a worse prognosis. In LUAD tissues, higher CD40LG expression was associated with longer Progression-Free Survival (PFS) and Overall Survival (OS). Moreover, CD40LG expression negatively correlated with the TNM stage and T stage in LUAD. Elevated CD40LG levels were linked to increased infiltration of CD8+ T cells. *In vitro* studies showed that modulating CD40LG affected LUAD cell metastasis and proliferation.

**Conclusion:** Our study demonstrates the pivotal role of CD40LG in LUAD, proposing its potential utility as a biomarker for prognosis and immunotherapy. The correlation between CD40LG expression, immune cell infiltration, and clinical outcomes emphasizes its importance in tumor-immune dynamics and the efficacy of immunotherapy.

## Introduction

With approximately 1.8 million deaths related to lung cancer, and 2.1 million new cases diagnosed each year, lung cancer is one of the most prevalent malignant tumor in the world 1^.^ Approximately 15% to 85% of cases of lung cancer fall into one of two primary categories: non-small-cell lung cancer (NSCLC) and small-cell lung cancer (SCLC). The most common type of NSCLC is LUAD (LUAD) 2, 3. Despite significant advances in diagnostic and therapeutic technology, survival rates for LUAD are disappointingly low, especially in metastatic cases. Currently, immunotherapy marks a significant breakthrough in cancer treatment and has demonstrated effectiveness across various cancers, including LUAD. However, the benefits of immunotherapy are not universal among LUAD patients, potentially owing to the immune microenvironment of the tumor. Therefore, there is a pressing necessity to identify specific immune-related molecules and novel immunotherapy targets for LUAD.

CD40LG, also known as CD154 or CD40 ligand, is a transmembrane protein mainly found on the surface of activated T cells. It is a key molecule in regulating immune response by interacting with its receptor, CD40, which is found on various immune cells 4, including dendritic cells 7, macrophages 6, and B cells 5. The primary function of CD40LG is to mediate T cell-dependent B cell activation 4, 5, which is essential for antibody production, germinal center formation, and the generation of memory B cells 5. Additionally, CD40LG plays a significant role in the activation and maturation of macrophages and dendritic cells, thereby influencing their ability to present antigens and secrete cytokines, which are vital for initiating and sustaining immune responses 6.

Recently, CD40LG has garnered significant attention within the oncology realm owing to its implication in shaping the tumor microenvironment. CD40LG has been detected in a variety of tumors and has the capability to influence the immune response by enhancing the activation and infiltration of immune cells like T cells, macrophages, and B cells 4-7. The expression of CD40LG has been linked to the enhancement of antitumor immunity through the stimulation of cytokine production and the promotion of cytotoxic T lymphocyte (CTL) responses 4. Research indicates that increased CD40LG expression correlates significantly with the infiltration of diverse immune cell subsets, such as CD8+ T cells, CD4+ T cells, dendritic cells, B cells, and natural killer (NK) cells, within tumor microenvironments. These results underscore the critical involvement of CD40LG in shaping the immune milieu of tumors 4-8. The expression of CD40LG also plays a crucial role in the efficacy of immunotherapy 8.

Additionally, CD40LG has been associated with controlling cell proliferation and oncogenic conversion, suggesting its promise as a therapeutic target 9. For example, research has indicated that the activation of CD40/CD40LG pathway has the potential to trigger apoptosis in tumor cells and impede tumor advancement via immune-mediated processes 10. In the sphere of LUAD research, investigations have underscored a noteworthy association between the expression of CD40LG and the immune microenvironment, suggesting that CD40LG might have a pivotal function in modulating immune reactions in LUAD 11. Despite these findings, the exact mechanisms through which CD40LG influences tumor development and immune interactions in NSCLC remain to be fully elucidated, necessitating further experimental studies to explore the potential therapeutic implications of targeting CD40LG in cancer.

In this investigation, we evaluated the expression of CD40LG in 94 samples of LUAD tissue alongside their respective adjacent non-cancerous tissues utilizing immunohistochemical staining techniques. Furthermore, the relationship between the infiltration of multiple immune cell populations and CD40LG expression was investigated, with a particular focus on elucidating the potential immunomodulatory function of CD40LG in LUAD. This was achieved through the performance of multiple immunofluorescence staining (mIF) on tissue microarrays. The objective of this research is to elucidate the significant role of CD40LG in NSCLC and to uncover the mechanisms through which CD40LG may influence tumor-infiltrating immune cells.

## Methods

### Data sources

The unified pan-cancer dataset was constructed by merging RNA-seq data from the unified pan-cancer dataset was constructed by merging RNA-seq data from TCGA and TARGET databases. Batch effects were minimized using ComBat algorithm and gene expression values were normalized to transcripts per million (TPM). Differential expression and clinical feature analyses were conducted using R software. We collected data from the Kaplan-Meier Plotter. This dataset encompasses prognostic information for patients undergoing immunotherapy across various cancer types 12. Gene expression and prognosis of patients were analysed through the GEO database using GSE118370, GSE43458, GSE30219, GSE31210 and GSE72094, it should be noted that only samples with an overall survival (OS) greater than 0 and a pathological type of LUAD in the above dataset were included in this study. CD40LG protein expression data for LUAD were downloaded from the Human Protein Atlas.

### Prognostic analysis

A thorough prognostic dataset sourced from a study based on TCGA was acquired 13. The CoxPH function in R was utilized to develop the Cox proportional hazards regression model. The optimal threshold for CD40LG expression was identified utilizing the MaxStat feature within the R programming environment, where the range for grouping was specified between the 25th and 75th percentiles. Each type of cancer was categorized into cohorts based on high and low levels of CD40LG expression. The survfit function in the R programming environment was employed to assess disparities in PFS and OS.

### Immune cell infiltration and immune score analysis

This investigation employed six different techniques to analyze tumor-infiltrating immune cells (TICs), utilizing algorithms available in the R package IOBR software package 14. These methods encompass TIMER 15, deconvo_EPIC 16, deconvo_MCPcounter 17, deconvo_xCell 18, deconvo_CIBERSORT 19, and deconvo_quanTIseq 20. Additionally, we examined the relationship between CD40LG expression and t immunophenoscore(IPS) using the deconvo_ips function methods 21,22.

### Lung adenocarcinoma tissue microchip and Immunohistochemistry

We acquired tissue microchips consisting of 94 pairs of LUAD tissues and Paracancerous tissues from Saville Biotech (Wuhan, China). Inclusion criteria included (1) no preoperative chemotherapy, radiotherapy, or immunotherapy; (2) radical lung cancer surgery (including lobectomy, composite lobectomy, sleeve resection, and total pneumo-nectomy) + systematic lymph node dissection; (3) confirmed diagnosis of adenocarcinoma of the lung; and (4) complete medical history. Each specimen was evaluated using a semi-quantitative scoring system (0-12) by multiplying the staining intensity score (0: negative; 1: weak; 2: moderate; 3: strong) by the percentage of positive cells (1: 1-25%; 2: 26-50%; 3: 51-75%; 4: 76-100%). Two blinded pathologists independently assessed all slides, with discordant cases (score difference >3) resolved through consensus review. Table 1 shows detailed demographic and clinical characteristics. The sections underwent deparaffinization and rehydration procedures initially, followed by antigen retrieval utilizing a sodium citrate buffer. Afterwards, they were incubated with the primary antibody against CD40LG (1:150, 16668-1-AP, Proteintech) at 4°C overnight. Following three washes in TBST, slides were treated with anti-rabbit IgG (Proteintech) and allowed to incubate at ambient temperature for one hour. Following the initial treatment with phosphate-buffered saline (PBS), the slides underwent a rinsing process, followed by diaminobenzidine treatment and counterstaining with hematoxylin. Each tissue sample underwent evaluation based on the intensity and coverage of immunohistochemical staining, employing a comprehensive scoring system ranging from 0 to 12. This system combines the staining intensity (rated as 0, 1, 2, or 3) with the staining extent (rated as 1, 2, 3, or 4) to provide a thorough assessment. Two pathologists, who were not aware of the clinicopathological information, conducted separate evaluations of the staining outcomes.

### Multiplex immunofluorescence

Yucebio Technology in Shenzhen, China, conducted multiplex immunofluorescence (mIF) staining and initial assessment. They processed 5 μm thick paraffin embedded tissue sections, dewaxed with xylene, rehydrated, and fixed. The slides were then stained with various markers and imaged using Vectra Polaris (Akoya Biosciences). Imaging was performed at 20× resolution (0.5 μm/pixel) using Vectra Polaris with spectral unmatching error <5%.

### Cell transfection

The cell lines, A549, H1975, PC9, H1299, and H1650, were acquired from the ATCC. For transfection experiments, Lipofectamine 2000 (Invitrogen, USA) was utilized to deliver either CD40LG-specific siRNA or a scrambled control siRNA (Ribobio). A549 cells were transfected with either pCDNA3.1-CD40LG (Biomed Gene, China) or pCDNA3.1 containing a non-targeting sequence using Lipofectamine 2000.

### Western blot

We commenced by extracting, quantifying, and subsequently separating protein samples on 10% agarose gels. Employing a semidry transfer system, we transferred these samples onto PVDF membranes. To mitigate nonspecific binding, the membranes underwent blocking with 5% milk at 25°C for 2h. Subsequently, they were incubated with the primary antibody against GAPDH (1:1,000, 60004-1-lg, Proteintech) and CD40LG (1:1,000, 16668-1-AP, Proteintech) at 4°C overnight. Then, membranes were probed with secondary antibodies (Thermo Fisher Scientific, diluted at 1:5,000) at 25°C for 1h. Visualization of protein bands were using Pierce ECL substrate (Thermo Fisher Scientific).

### Transwell assay

In migration experiment, A549 and H1975 were introduced on the upper chamber in a serum-free medium, while the lower chamber was supplied with 10% serum medium. A549 and H1975 that traversed to the lower chamber were immobilized in 4% paraformaldehyde and stained with crystal violet after a 24-hour incubation period. Transwell chambers were precoated with Matrigel firstly in invasion assays.

### Scratch wound-healing assay

The experiment commenced with the inoculation of cells into 6-well plates, where they were nurtured until reaching confluency. Subsequently, a controlled wound was induced utilizing a pipette tip, followed by a thorough cleansing of the cellular milieu with PBS. Then, the cells were subjected to an environment devoid of serum, and the progression of wound closure was meticulously observed at distinct intervals employing microscope.

### Statistical analysis

The Wilcoxon Tests were utilized for unpaired data. The Kruskal-Wallis test was utilized for multiple groups data. Pearson's correlation coefficient was employed to ascertain correlations, and survival analysis was conducted employing the Log-rank test. A *P* ≤ 0.05 was considered statistically meaningful.

## Results

### Down-Regulation of CD40LG in Multiple Cancer Types and Its Prognostic Value

**Figure 1:**
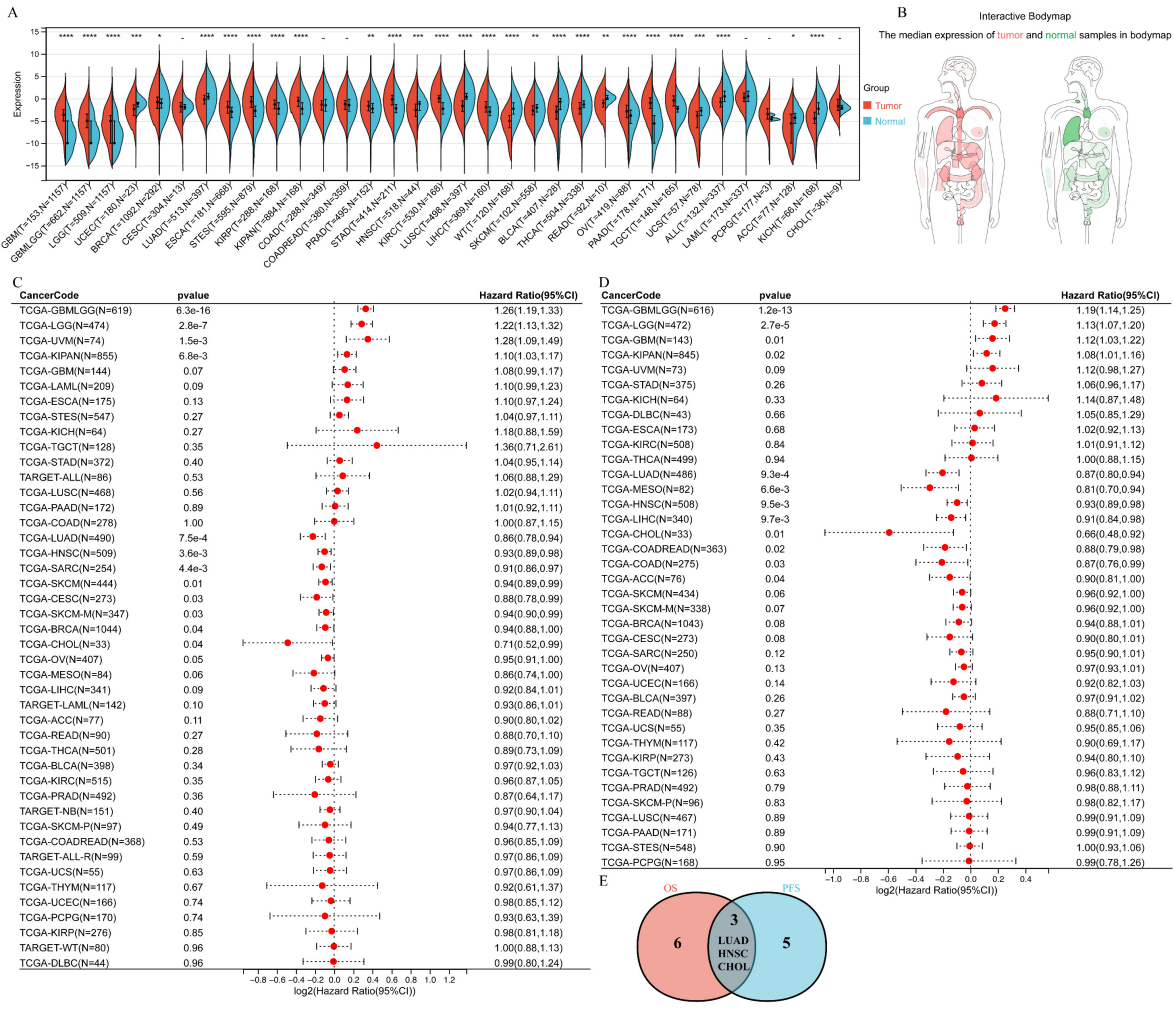
** CD40LG is down-regulated in multiple cancer types, and this down-regulation has significant prognostic implications. (A)** Violin plots showed the differential expression of CD40LG in 34 cancer types compared with normal tissues. **(B)** Body map showing CD40LG expression levels in various normal human tissues. **(C)** Cox regression analysis of OS demonstrated the prognostic role of CD40LG in a variety of cancers. **(D)** Cox regression analysis of PFS demonstrated the prognostic role of CD40LG in a variety of cancers. **(E)** The veen diagram shows those cancers in which CD40LG has the same prognostic predictive efficacy in OS and PFS.

In 14 out of 34 cancer types analyzed, CD40LG exhibited significant down-regulation in tumor specimens, including KICH, ACC, UCEC, SKCM, LUAD, READ, BLCA, LUSC, WT, THCA, READ, UCS, ALL, and HNSC (Figure 1A). In the course of our investigation using the interactive body map, a notable observation emerged: the level of CD40LG expression in the normal human lung surpassed that of various other organs, notably including lung tumors (Figure 1B). Utilizing Cox regression analysis to evaluate overall survival, we observed a favorable prognosis associated with elevated CD40LG expression across nine cancer types, namely LUAD, HNSC, SARC, and CHOL. Conversely, poor prognostic outcomes were noted in only four cancer types, namely GBMLGG, LGG, KIPAN, and UVM (Figure 1C). Cox regression analysis showed a significant relationship between higher CD40LG expression and improved prognosis across eight cancer types, namely LUAD, HNSC, LIHC, and CHOL. Conversely, poor prognosis was observed in only four cancer types, namely GBMLGG, LGG, GBM, and KIPAN (Figure 1D). In LUAD, CHOL, and HNSC, elevated levels of CD40LG were correlated with better PFS and OS (Figure 1E). The correlations for the aforementioned three tumors were reaffirmed through application of the log-rank test, as illustrated in Supplementary Figure 1.

Decreased expression of CD40LG also indicated higher TNM stage in LUAD and KIRP (Supplementary Figure 2A), higher T stage in LUAD, and HNSC (Supplementary Figure 2B). In KIPAN, STEC, LGG, SARC, ESCA, GBMLGG, LAML, LUAD, and READ, the expression level of CD40LG was positively correlated with age (Supplementary Figure 2C). Notably, LUAD exhibited the strongest correlation coefficient in the analysis of CD40LG expression and age (Supplementary Figure 2C). Male patients in LUAD, BRCA, STES, STAD, LUSC, and BLCA presented significantly lower CD40LG expression than females (Supplementary Figure 2D).

### Exploring the Relationship Between CD40LG Expression and Immunotherapeutic Response Across Diverse Cancer Types

**Figure 2:**
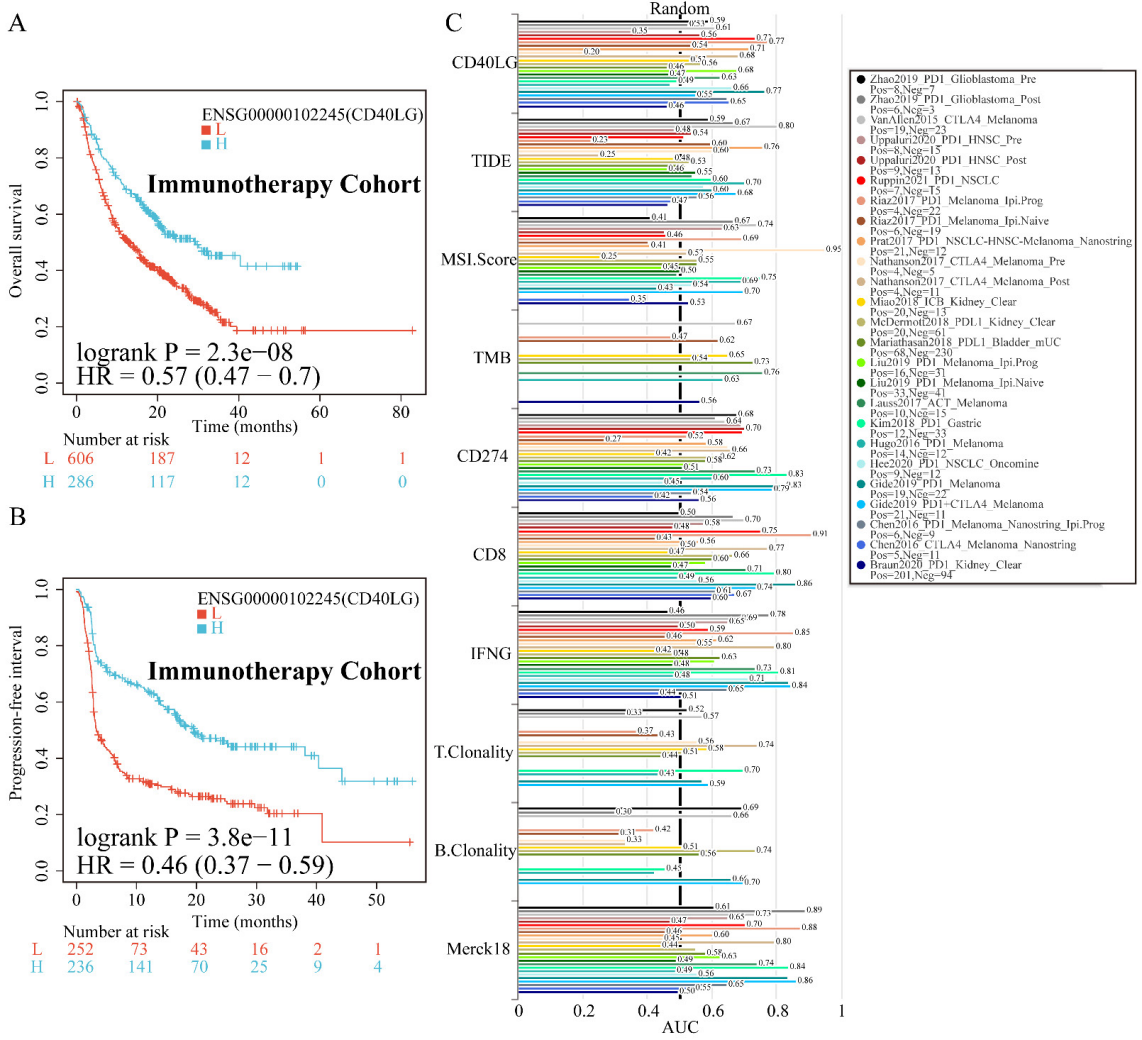
** Correlation Between CD40LG Expression and Immunotherapeutic Efficacy of Various Cancers. (A)** Kaplan-Meier plotter analysis showing that patients with high CD40LG expression have better OS after immunotherapy.** (B)** Kaplan-Meier plotter analysis showing that patients with high CD40LG expression have better PFS after immunotherapy.** (C)** Comparison of CD40LG expression with traditional biomarkers including PD-L1 expression, TMB, MSI status and CD8+T cell density in predicting immunotherapy outcomes across 25 datasets.

We obtained prognostic data for patients undergoing immunotherapy from the Kaplan-Meier plotter database. The subjects were categorized according to varying levels of CD40LG expression. The findings consistently demonstrate that individuals exhibiting elevated CD40LG expression within tumor tissues exhibited significantly improved prognoses in terms of both OS (HR=0.57, P <0.0001, Figure 2A) and PFS (HR=0.46, P<0.0001, Figure 2B). In 25 immunotherapy datasets, we compared CD40LG expression with traditional biomarkers used to predict immunotherapy outcomes. We found that CD40LG expression demonstrated superior efficacy in predicting patient prognosis following immunotherapy, surpassing many conventional biomarkers (Figure 2C). Notably, in the Ruppin2021_PD1_NSCLC dataset, the AUC value for ROC analysis of CD40LG expression reached 0.73, second only to CD8 expression at 0.75 and significantly higher than other indicators (Figure 2C).

### The important role of CD40LG expression in lung adenocarcinoma

Drawing from the findings delineated above, it is discernible that the manifestation of CD40LG in LUAD evinces significant associations with patient prognosis and clinical staging. Moreover, its presence bears relevance to patient prognostication subsequent to immunotherapeutic interventions. Therefore, we will focus on the role of CD40LG expression in LUAD.

**Figure 3:**
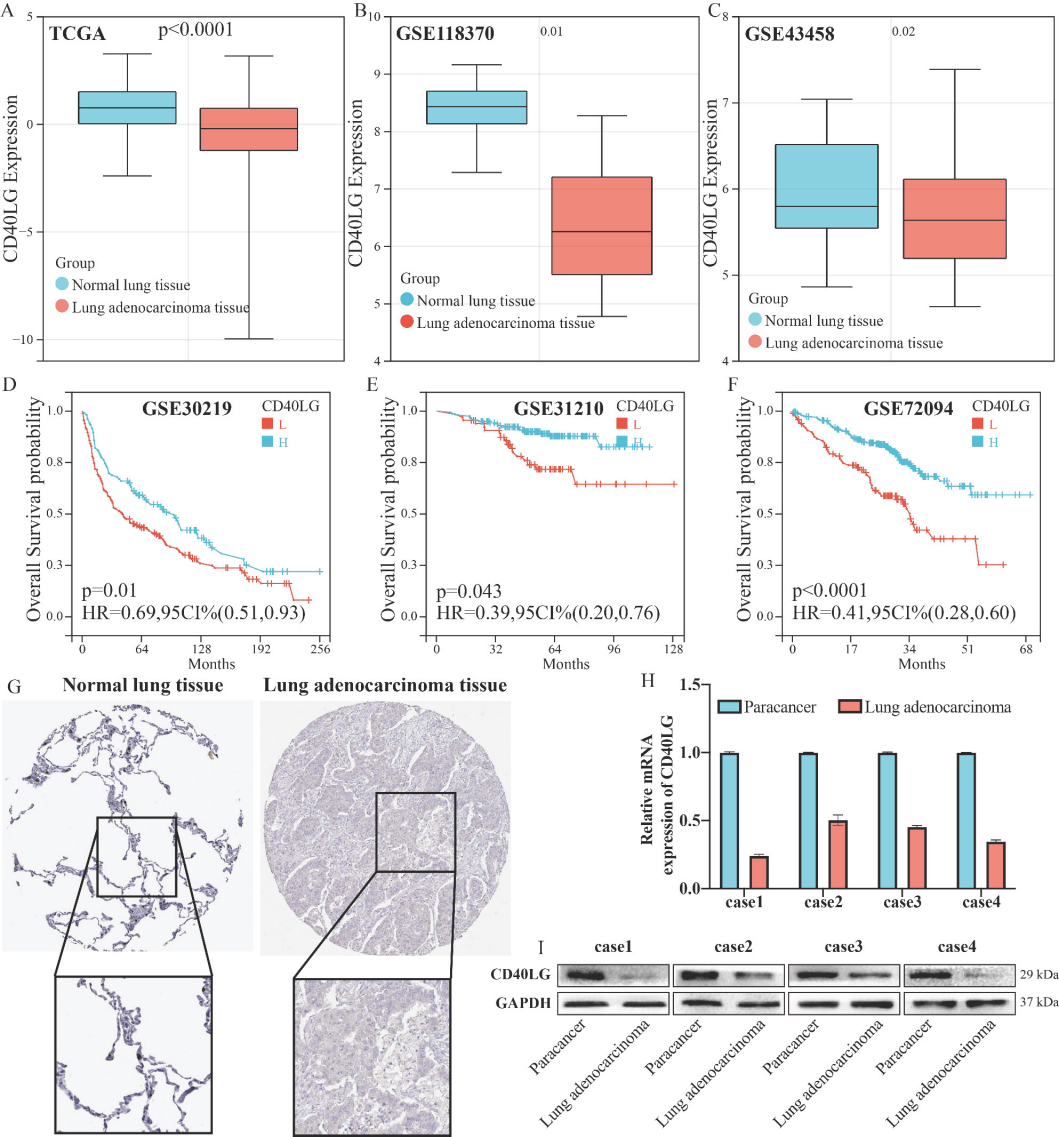
** The important role of CD40LG expression in LUAD. (A-C)** Reduced expression of CD40LG in LUAD was analysed in public databases, including TCGA, GSE118370 and GSE43458.** (D-F)** The association of CD40LG expression with overall survival of LUAD patients was examined in public databases, including GSE30219, GSE31210 and GSE72094.** (G)** CD40LG expression in LUADs analysed in immunohistochemistry of HPA was lower than in corresponding paraneoplastic tissues. **(H)** PCR experiments were performed on four patients with LUAD to verify the RNA expression of CD40LG in cancer and corresponding paracancerous tissues. (I) Western blot experiments were performed on four patients with LUAD to verify the protein expression of CD40LG in cancer and corresponding paracancerous tissues.

In our analysis of several public databases, including TCGA (Figure 3A), GSE118370(Figure 3B), and GSE43458(Figure 3C), we discovered that CD40LG expression is significantly higher in paracancerous tissues compared to LUAD tissues. Next, we verified through multiple public databases, including GSE30219(Figure 3D), GSE31210(Figure 3E), and GSE72094(Figure 3F), that higher CD40LG expression in LUAD is associated with better patient prognosis. The immunohistochemistry of CD40LG obtained from HPA also suggested that the expression of CD40LG was higher in paracancerous tissues compared with LUAD tissues (Figure 3G). PCR and Western blot experiments conducted on four LUAD tissues and their adjacent normal lung tissues confirmed that CD40LG expression was higher in the adjacent normal tissues at both the transcript (Figure 3H) and protein (Figure 3I) levels compared to the adenocarcinoma tissues.

### CD40LG expression correlates with LUAD immune microenvironment

**Figure 4:**
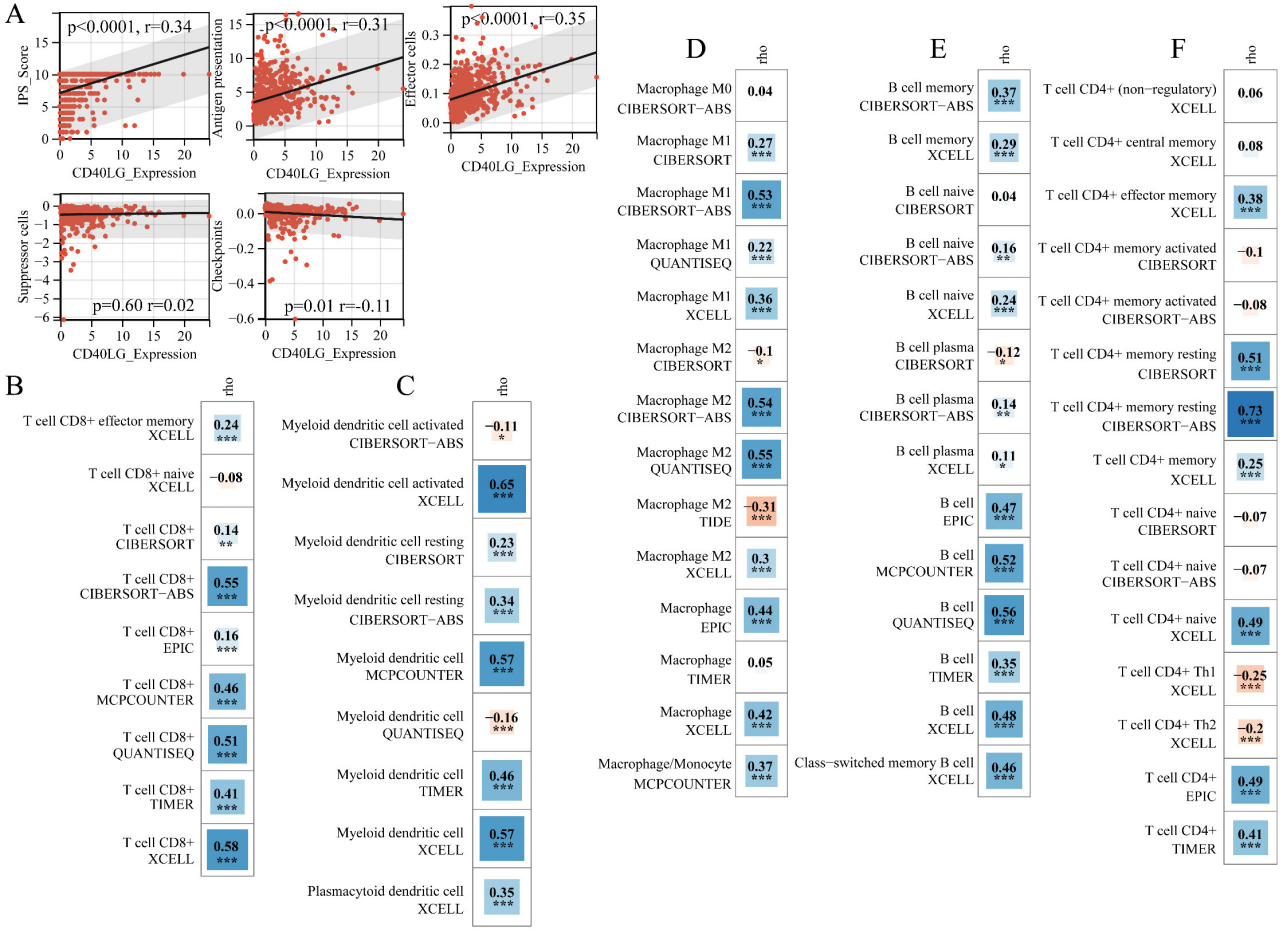
** CD40LG Expression Correlates with Immune Microenvironment in LUAD. (A)** Correlation between CD40LG expression and Immunophenoscore (IPS), including components such as antigen presentation, effector cells, suppressor cells, and immune checkpoints in LUAD.** (B-F)** Positive correlation of CD40LG expression with infiltration of various immune cells in LUAD tissues, including CD8+ T cells, dendritic cells, macrophages, B cells, and CD4+ T cells, using multiple predictive methods.

In the TCGA database for LUAD, we calculated the Immunophenoscore (IPS) for patients. The immune priming system (IPS) is comprised of four essential elements: MHC molecules, which are represented antigen presentation; suppressor cells (SC), which regulate immune reactions; effector cells (EC), which execute immune responses; and immune checkpoints (CP), which modulate immune activity. The IPS-Score serves as an indicator of tumor immunogenicity. Our study shows a positive correlation between IPS score and CD40LG expression (Figure 4A). Antigen presentation is positively correlated with CD40LG expression (Figure 4A). There is a similar relationship between the effector cell function and CD40LG expression also (Figure 4A). On the flip side, there was an inverse association observed between CD40LG and immune checkpoint expression (Figure 4A). We employed eight distinct methodologies to prognosticate the infiltration levels of effector and antigen-presenting cells within TCGA LUAD specimens. These methodologies encompassed the prediction of dendritic cells, CD8+ T cells, B cells, macrophages, and CD4+ T cells. The results showed a positive association between CD8+ T cells infiltration and CD40LG expression significantly, as evidenced by five distinct analytical approaches (Figure 4B). Furthermore, we found a consistent positive correlation between dendritic cell infiltration and CD40LG expression across four different methodologies (Figure 4C). In the same vein, CD40LG expression exhibited notable positive correlation with macrophage infiltration across five distinct methodologies (Figure 4D). Furthermore, CD40LG expression displayed a significant positive association with B cell infiltration across the same five methodologies, with all correlation coefficients exceeding 0.4 (Figure 4E). Finally, a significant positive association was found between CD4+ T cells infiltration and CD40LG expression across five analytical approaches, with correlation coefficients exceeding 0.4 (Figure 4F). Consequently, our subsequent investigations will focus on validating this correlation by examining macrophages, B cells, CD8+ T cells, and CD8+ T cells CD4+ T cells.

### IHC verification of CD40LG expression in lung adenocarcinoma tissues

**Figure 5:**
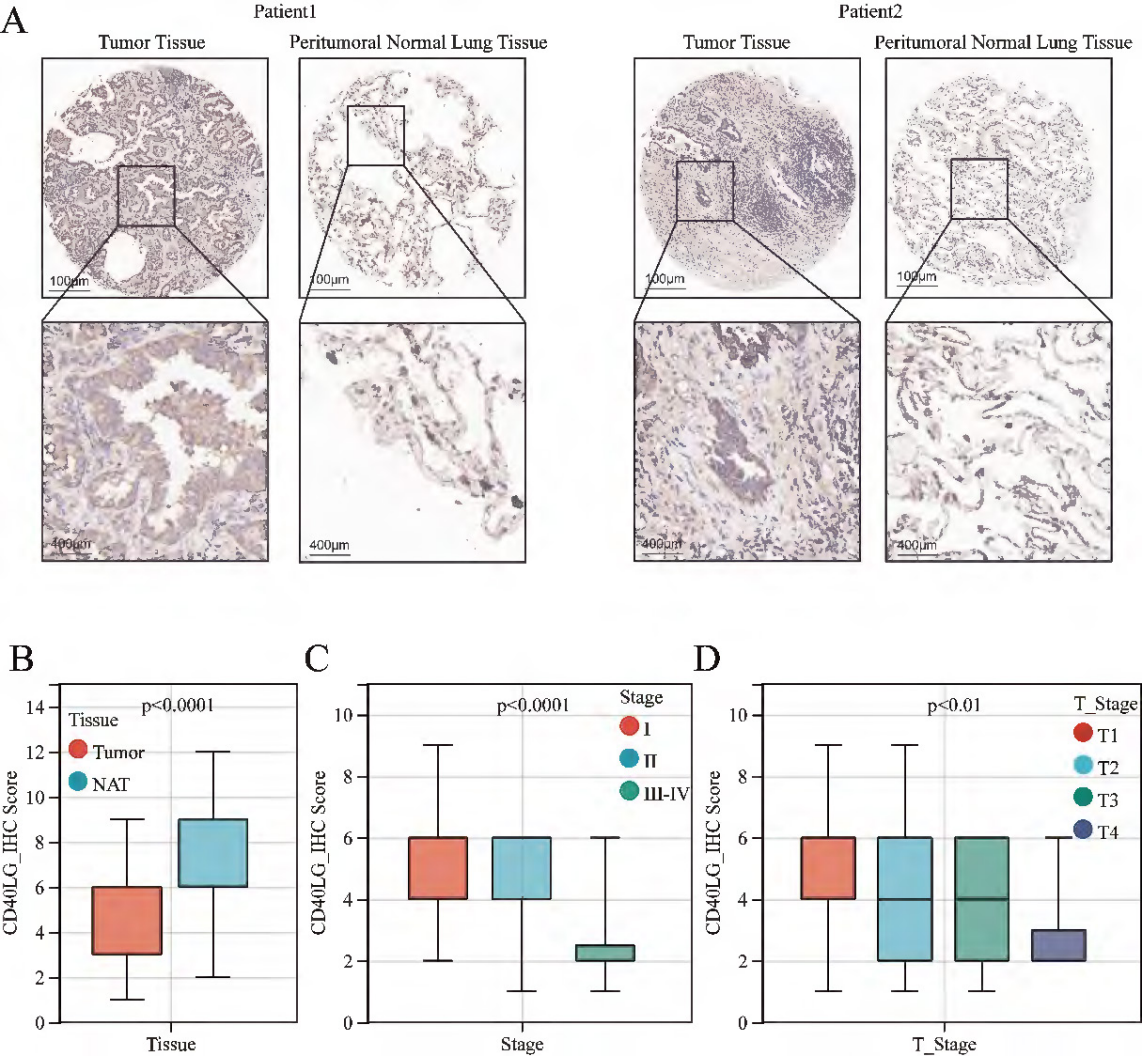
** CD40LG Expression in LUAD and its Association with Clinical Stages. (A)** Representative immunohistochemical staining images of CD40LG in primary LUAD tissues and corresponding paracancerous tissues.** (B)** Boxplot comparing CD40LG expression levels in tumor tissues versus paracancerous tissues.** (C-D)** The boxplot analysis illustrates the expression of CD40LG in relation to TNM and T stages.

For additional validation, we procured tissue microarrays comprising 94 pairs of LUAD tissues alongside their corresponding paracancerous tissues. Comprehensive clinical baseline information is detailed in Table 1. The cohort predominantly comprised Asian patients (100%) with stage I disease (61.7%), reflecting the demographic characteristics of the tertiary cancer center. IHC results revealed that CD40LG is mainly distributed on the plasma membrane and cell membrane, presenting patchy or clustered distribution (Figure 5A). The median expression level of CD40LG divided patients into two groups: the CD40LG high expression group and the CD40LG low expression group. Table 2 shows the CD40LG analysis results stratified by each clinical factor. Boxplot analysis shows that the expression of CD40LG is significantly increased in paracancerous tissues compared to cancer tissues (P<0.0001, Figure 5B), corroborating our previous bioinformatics analysis findings. Moreover, decreased CD40LG expression correlated with advanced TNM stage (P<0.0001, Figure 5C) and T stage (P<0.01, Figure 5D), which corroborate our previous bioinformatics analysis.

### CD40LG expression was correlated with LUAD immune cell infiltration

**Figure 6:**
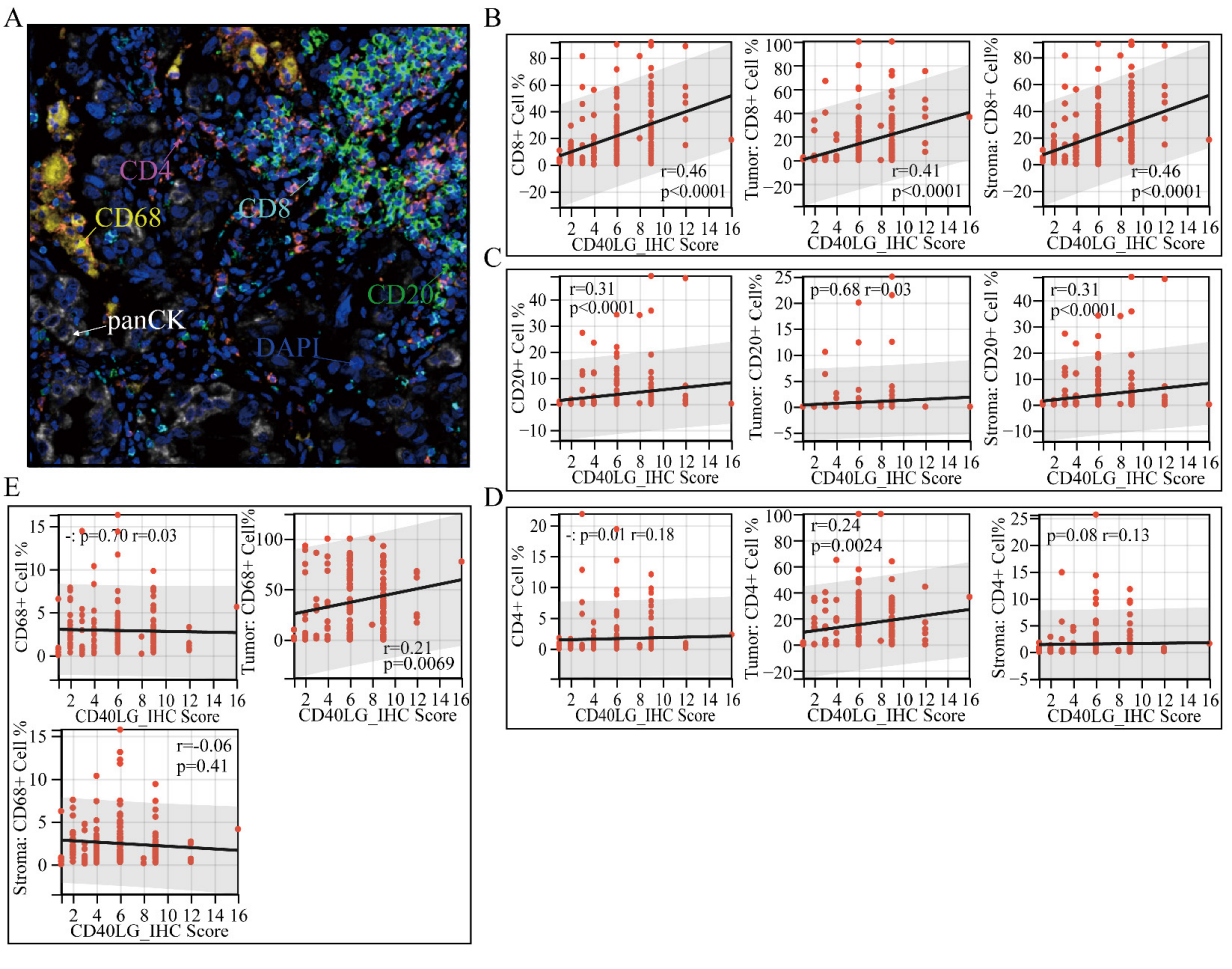
** CD40LG Expression Correlated with Immune Infiltrate Patterns in LUAD. (A)** Representative mIF staining image showing CD40LG expression and immune cell distribution in LUAD tissue microarrays.** (B-E)** Correlation analysis between CD40LG_IHC_Score and percentages of various immune-related cells in total, tumor, and stromal regions.

To investigate the associtation between immune cell infiltration and CD40LG expression in greater detail, we employed multiplex immunofluorescence (mIF) technique to identify the presence of specific immune cells in tissue microarrays. Table 3 outlines the specific antibodies and the corresponding cells they recognize. Figure 6A illustrates a typical example of mIF staining. The median expression level of CD40LG divided patients into two groups: the CD40LG high expression group and the CD40LG low expression group. Tumors with elevated CD40LG expression were significantly more infiltrated by CD20+ B cells, CD4+ T cells, CD68+ macrophages, and CD8+ T cells (Table 4).

Furthermore, using panCK staining, we divided the tissues into total, tumor, and stromal regions, and conducted a correlation analysis between the percentage of immune cells and CD40LG_IHC_Score in each region. A positive correlation was observed between the CD40LG IHC score and the proportion of CD8+T cells in the total region, cancer region, and stromal region. All p values are less than 0.0001, and the correlation coefficients are greater than 0.4 (Figure 6B). There is a significant positive correlation between CD20+B cells infiltration and CD40LG IHC Score in both the total and stromal regions, with a correlation coefficient exceeding 0.3 and a p-value below 0.0001 (Figure 6C). Regarding CD4+ T cells, the CD40LG_IHC_Score showed a weaker positive correlation only in the total regions (r = 0.18, p = 0.01) and stromal regions (r = 0.24, p = 0.0024) (Figure 6D). Lastly, for CD68+ macrophages, the CD40LG_IHC_Score was weakly positively correlated only in the tumor region (r = 0.21, p = 0.0069) (Figure 6E).

### CD40LG expression is linked to the proliferation and migration of LUAD cells

**Figure 7:**
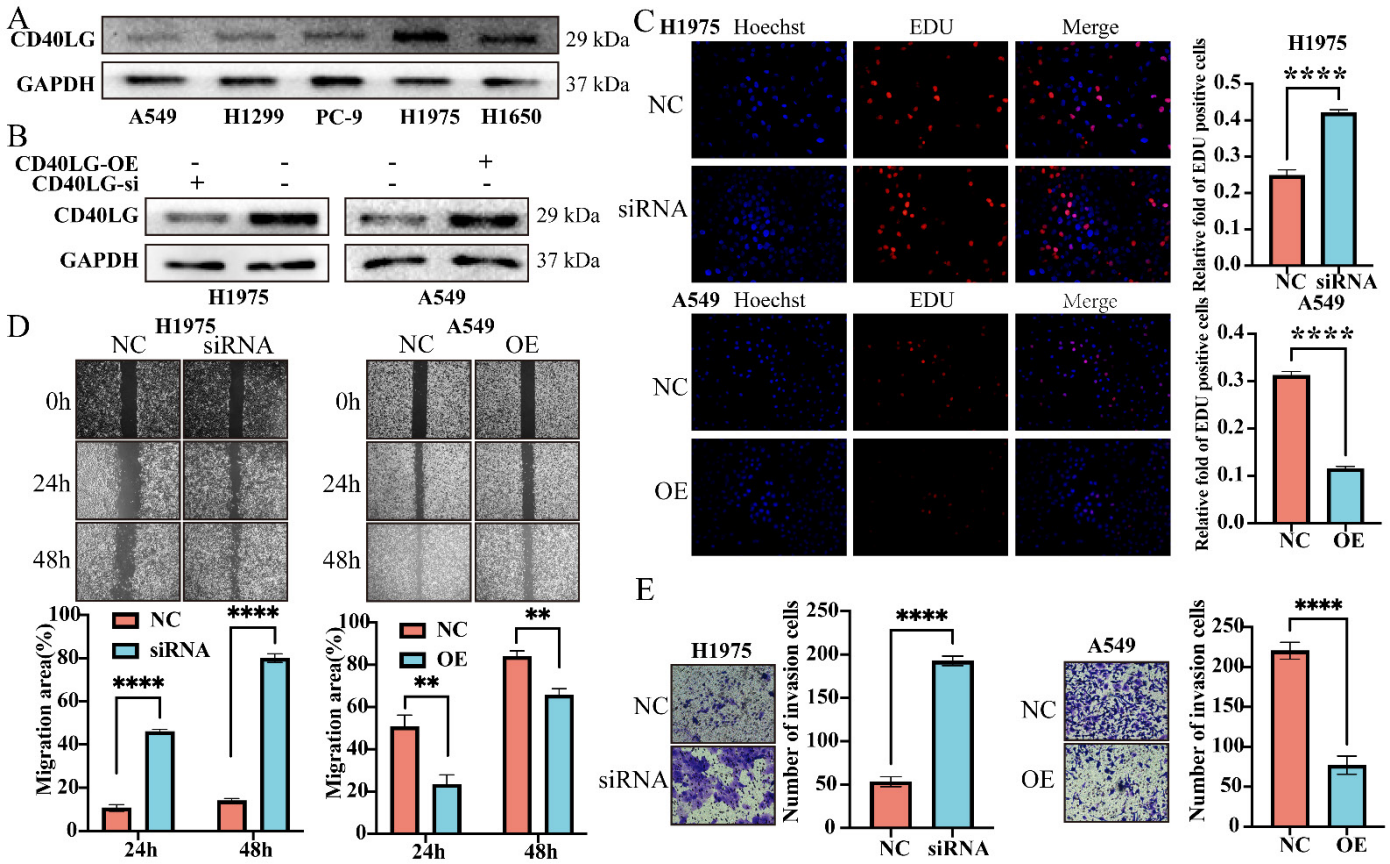
** CD40LG Expression and Its Effects on LUAD Cell Migration, Invasion, and Proliferation. (A)** Western blot analysis of CD40LG expression in five LUAD cell lines.** (B)** Western blot verification of siRNA-mediated knockdown of CD40LG in H1975 cells and overexpression in A549 cells.** (C)** EDU assay results showing increased proliferation upon CD40LG knockdown in H1975 cells and decreased proliferation with CD40LG overexpression in A549 cells.** (D)** Scratch wound healing assay indicating enhanced migration with CD40LG knockdown in H1975 cells and reduced migration with CD40LG overexpression in A549 cells.** (E)** Transwell assay demonstrating increased migration and invasion capabilities with CD40LG knockdown in H1975 cells and decreased capabilities with CD40LG overexpression in A549 cells.

To further analyze the impact of CD40LG on LUAD cells, we examined CD40LG protein expression level in five LUAD cell lines, including A549, H1975, PC-9, H1299, and H1650, using Western blot analysis. Our analysis revealed that H1975 cells exhibited the highest CD40LG expression, while A549 cells showed the lowest levels (Figure 7A). To further investigate, we knocked down CD40LG in H1975 cells and induced overexpression of CD40LG in A549 cells, with confirmation achieved via Western blot analysis (Figure 7B).

Through the EDU assay, we discovered that knocking down CD40LG in H1975 cells significantly enhanced their proliferative capacity, whereas overexpressing CD40LG in A549 cells significantly reduced their proliferative capacity (Figure 7C). In the scratch wound healing assay, we observed that knocking down CD40LG in H1975 cells significantly increased their migratory ability, while overexpressing CD40LG in A549 cells significantly decreased their migratory ability (Figure 7D). Similar results were obtained from the Transwell assay: knocking down CD40LG in H1975 cells significantly enhanced their migration and invasion capabilities, whereas overexpressing CD40LG in A549 cells significantly reduced their migration and invasion capabilities (Figure 7E).

## Discussion

In our investigation, our study revealed that higher CD40LG expression inversely correlated with higher TNM stages and T stages in LUAD. This observation suggests that decreased CD40LG levels may contribute to tumor progression, aligning with previous reports that link lower CD40LG expression to more advanced cancer stages and poor prognoses 11. CD40LG exhibits different prognostic effects in different types of cancer, which may reflect tissue-specific immune environments. In gliomas, overexpression of CD40LG may promote activation of immunosuppressive microglia, while in LUAD, it enhances recruitment of cytotoxic T cells. The negative correlation observed between CD40LG levels and disease severity highlights its potential as a biomarker for measuring disease staging and progression.

Our research also highlights the association of high CD40LG expression with improved responses to immunotherapy, outperforming traditional biomarkers in predictive efficacy. This finding is particularly noteworthy given the current emphasis on immune checkpoint blockade therapies. Previous research has suggested that CD40LG has the potential to augment the effectiveness of immunotherapy by altering the immune microenvironment in a manner conducive to eliciting anti-tumor reactions 4-7. Our data corroborate these reports and suggest that CD40LG could be instrumental in-patient stratification and personalized treatment approaches for NSCLC.

Furthermore, our findings indicated that CD40LG protein level was closely associated with multiple immune cell infiltration, including macrophages, CD4+ T cells, and CD8+ T cells. Notably, CD8+ T cells infiltration exhibited the strongest correlation with CD40LG expression, with the highest correlation coefficient observed in this regard. These findings align with prior studies indicating that CD40LG is crucial in modulating the immune response within the tumor microenvironment. For example, prior studies have elucidated the capacity of CD40LG to augment immune response through its promotion of immune cell infiltration and activation, as well as its stimulation of cytokine synthesis. These mechanisms collectively support the elicitation of cytotoxic T lymphocyte (CTL) reactions 4-8. Our results provide further support for this mechanism, showing positive correlations between CD40LG expression and immune cell infiltration. Additionally, our correlation investigation concerning CD40LG_IHC_Score and the proportions of immune-related cells across diverse tissue zones unveiled a robust affirmative correlation with CD20+ B cells and CD8+ T cells. This finding is suggesting that CD40LG promotes the presence and activity of key immune effector cells within the tumor microenvironment 4-7. CD40LG's interaction with its receptor CD40 on various immune cells likely enhances the overall antitumor immune response, contributing to the observed correlations between CD40LG expression and improved clinical outcomes.

*In vitro* functional assays demonstrated that CD40LG knockdown in H1975 cells significantly enhanced their proliferative, migratory, and invasive capabilities, while CD40LG overexpression in A549 cells had the opposite effect. This indicates that CD40LG not only modulates the immune microenvironment but also directly influences tumor cell behavior.

In summary, our investigation delineates the intricate functions of CD40LG within the context of LUAD, highlighting its importance in both immune modulation and direct tumor cell behavior. Compared to PD-L1 IHC (current gold standard), CD40LG quantification showed comparable inter-observer concordance. However, the cost-effectiveness of multiplex CD40LG assessment needs evaluation against existing biomarker panels. While our study leveraged bulk RNA-seq data and advanced deconvolution algorithms to characterize immune infiltration, these approaches cannot capture the spatial organization of immune cells within tumor microenvironment. Emerging spatial transcriptomics technologies may provide complementary insights into CD40LG's role in orchestrating immune cell localization. Future research should focus on further elucidating the mechanistic pathways through which CD40LG exerts its effects and exploring its potential in clinical applications for LUAD treatment.

## Supplementary Material

Supplementary figures.

## Data and materials availability

The present study utilized and analyzed data sourced from the TCGA and GEO databases. For further inquiries regarding this research, please reach out to the corresponding author. All requisite data for evaluating the outcomes are provided within this manuscript.

## Figures and Tables

**Table 1:** Clinical characteristics of patients with LUAD tissue microarrays.

Characteristics	LUAD(N=94)
Age	
<65	63(67.02%)
>=65	31(32.98%)
Gender	
Female	62(65.96%)
Male	32(34.04%)
Stage	
I	58(61.70%)
II	17(18.09%)
III-IV	19(20.21%)

**Table 2:** Immunohistochemical results of CD40LG immunohistochemistry of LUAD tissue microarrays and their relationship with clinic pathophysiological factors.

Characteristics	CD40LG		Total	P value
	Low(N=46)	High(N=48)	(N=94)	
Age				0.46
<65	33(35.11%)	30(31.91%)	63(67.02%)	
>=65	13(13.83%)	18(19.15%)	31(32.98%)	
Gender				1
Female	30(31.91%)	32(34.04%)	62(65.96%)	
Male	16(17.02%)	16(17.02%)	32(34.04%)	
Stage				<0.0001
I	16(17.02%)	42(44.68%)	58(61.70%)	
II	12(12.77%)	5(5.32%)	17(18.09%)	
III-IV	18(19.15%)	1(1.06%)	19(20.21%)	

**Table 3:** Multichromatic Immunofluorescent Staining of Antibodies and Their Corresponding Cellular Targets.

Color	Antibodies	Cells/Components
Opal-520	CD20	B-cell
Opal-620	CD4	Helper T-cell
Opal-480	CD8	Cytotoxic T-cell
Opal-570	CD68	Macrophage
Opal-780	PanCK	Tumor epithelial cell
DAPI	DAPI	Nucleus

**Table 4:** IHC assessment of CD40LG expression in lung cancer tissue microarrays and its correlation with the staining patterns of diverse tumor tissue markers.

Characteristics	CD40LG	
	Low(N=46)	High(N=48)
CD8+ Cell %		
Mean±SD	8.03±9.17	11.56±10.66
Median[min-max]	4.94[0.09,45.30]	8.91[0.69,47.86]
CD20+ Cell %		
Mean±SD	1.65±3.67	2.36±4.19
Median[min-max]	0.13[0.0e+0,12.44]	0.70[0.0e+0,21.75]
CD68+ Cell %		
Mean±SD	3.22±3.10	4.07±3.57
Median[min-max]	2.35[0.02,14.42]	3.20[0.34,16.28]
CD4+ Cell %		
Mean±SD	1.60±3.81	3.07±4.18
Median[min-max]	0.34[0.0e+0,21.82]	1.28[0.0e+0,19.36]
