# Supplementary material for: CD40LG Downregulation in Lung Adenocarcinoma: A Prognostic Biomarker Linked to Immune Cell Infiltration and Survival Outcomes
Source: J Cancer. 2025 Aug 22;16(13):3884–96. doi: 10.7150/jca.115525 (PMC12491049; doi:10.7150/jca.115525)
Supplement: Supplementary file 1 — Supplementary figures. [file jcav16p3884s1.pdf]

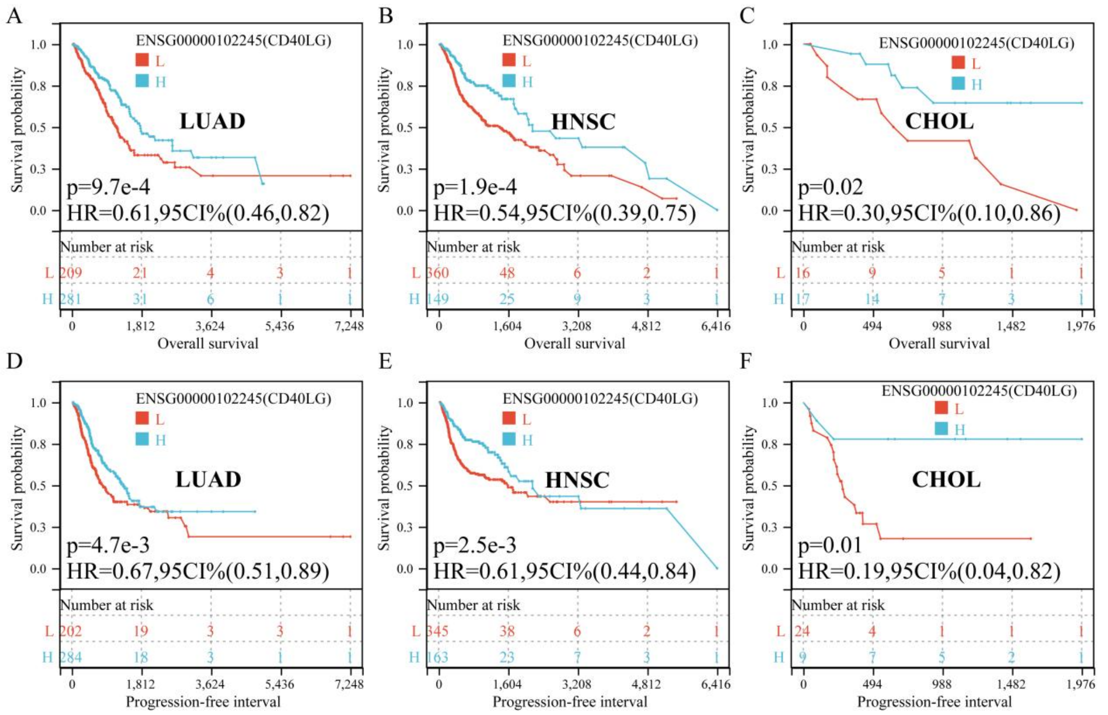

Supplementary Figure 1: Kaplan-Meier survival curves reconfirming the positive correlation between high CD40LG expression and better OS in LUAD(A), CHOL(B), and HNSC(C) and PFS in LUAD(D), CHOL(E), and HNSC(F).

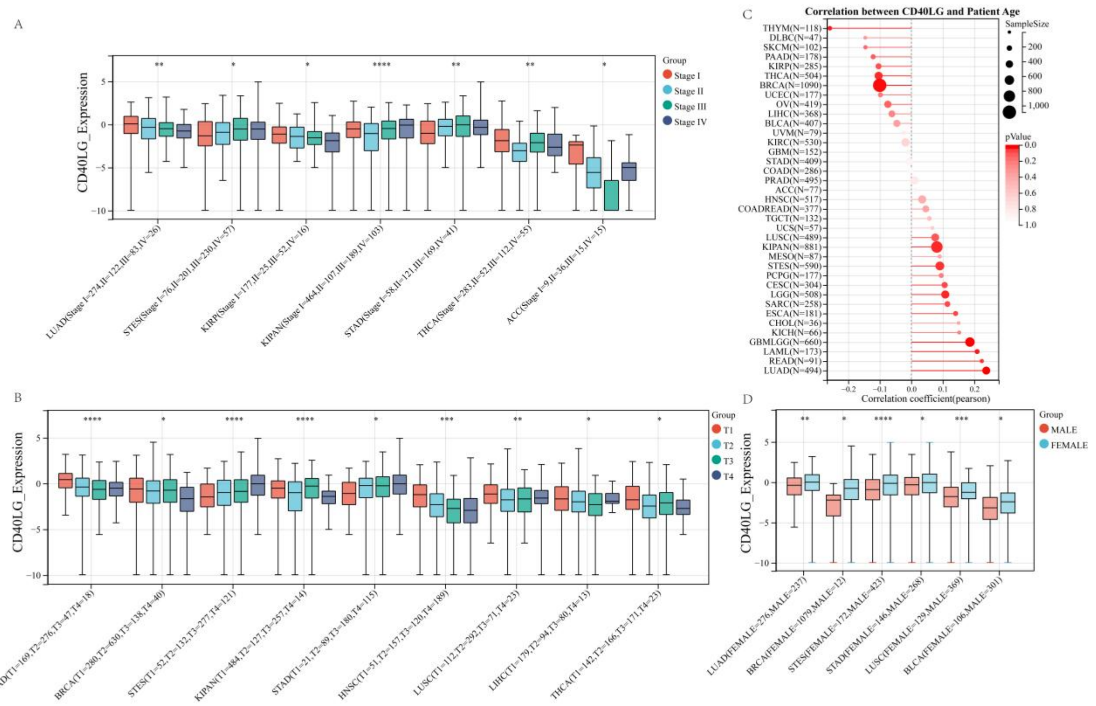

Supplementary Figure 2: Correlation of CD40LG Expression with Clinical Stages, Age, and Gender in Various Cancers. (A) CD40LG expression levels across different TNM stages. (B) CD40LG expression across different T stages. (C) Correlation analysis between CD40LG expression and age in multiple cancer types.(D)

Comparison of CD40LG expression between male and female patients in various cancers.
